# Development and assessment of an educational video to promote self-efficacy in newborn care

**DOI:** 10.1590/0034-7167-2025-0101

**Published:** 2025-12-12

**Authors:** Rhaiany Kelly Lopes de Oliveira, Maria Jocelane Nascimento da Silva, Hévila Ferreira Gomes Medeiros Braga, Emília Soares Chaves Rouberte, Flávia Paula Magalhães Monteiro, Antônio Marcos de Souza Soares, Benedita Shirley Carlos Rosa, Maria Vera Lúcia Moreira Leitão Cardoso, Emanuella Silva Joventino Melo

**Affiliations:** I Universidade Federal do Ceará. Fortaleza, Ceará, Brazil; II Universidade da Integração Internacional da Lusofonia Afro-Brazileira. Redenção, Ceará, Brazil

**Keywords:** Infant, Newborn, Self Efficacy, Validation Study, Audiovisual Aids, Nursing., Recién Nacido, Autoeficacia, Estudio de Validación, Recursos Audiovisuales, Enfermería.

## Abstract

**Objectives:**

to develop and assess an educational video to promote self-efficacy among newborn caregivers.

**Methods:**

a video development study conducted in five stages: content delimitation (narrative review and situational diagnosis); script content construction and validity (experts); video development; produced video validity (experts); and face validity (target audience). The Content Validity Index was used to analyze the data.

**Results:**

the video “*Cuidando do seu bebê*” is 24 minutes long and covers the main home care practices for newborns. The assessment was carried out from the perspective of appearance and content, presenting a Content Validity Index higher than 0.80 in all validity stages. The target audience unanimously considered the video relevant and clear.

**Conclusions:**

the video is valid for use by healthcare professionals as a tool to promote the self-efficacy of newborn caregivers.

## RESUMEN

**Objetivos:**

desarrollar y evaluar un video educativo para promover la autoeficacia en cuidadores de recién nacidos.

**Métodos:**

estudio del desarrollo del video en cinco etapas: delimitación del contenido (revisión narrativa y diagnóstico situacional); construcción y validación del contenido del guion (expertos); desarrollo del video; validación del video producido (expertos); y validación facial (público objetivo). Se utilizó el Índice de Validez de Contenido para analizar los datos.

**Resultados:**

el video “Cuidando do seu bebê” tiene una duración de 24 minutos y abarca las principales prácticas de cuidado domiciliario para recién nacidos. La evaluación se realizó desde la perspectiva de la apariencia y el contenido, presentando un Índice de Validez de Contenido superior a 0,80 en todas las etapas de validación. El público objetivo consideró unánimemente que el video era relevante y claro.

**Conclusiones:**

el video es válido para profesionales de la salud como herramienta para promover la autoeficacia de los cuidadores de recién nacidos.

## INTRODUCTION

The birth of a child causes the entire family nucleus to undergo transformations, turning its full attention to the child^(1)^. This event produces a mix of emotions and difficulties in family members as they adapt to their new routine, requiring the development of skills and safety in caring for newborns (NBs)^(2,3)^.

It is also known that the neonatal component prevails with the largest share of deaths with regard to overall infant mortality. According to data released by the United Nations Fund, in 2022, around 2.3 million infant deaths occurred in the first month of life^(4)^. According to the Ministry of Health, in 2019, Brazil recorded an alarming rate of 67.9 infant deaths per 1,000 live births, with emphasis on the North (16.6) and Northeast (15.2) regions^(5)^.

This alarming health reality is transforming the approach to nursing practice, as nurses are challenged daily to focus on health promotion and community education. This shift goes beyond the traditional approach focused on restorative care for patients with acute health problems, extending to areas such as child health in the primary care setting^(6)^.

Therefore, it is important to improve health education actions, providing comprehensive guidance and disseminating information during meetings between pregnant women and healthcare professionals^(7)^ as well as postpartum women and NBs’ family members. In this context, health education strategies that treat the mother and family as the primary caregivers of NBs are essential, especially at home.

Mothers need to believe in their ability to care for children so that they can actually carry out such care effectively. This belief is called self-efficacy, characterized as confidence in one’s ability to successfully perform behaviors related to parenting, playing a central role in this challenging scenario^(8)^. To this end, it should be considered that health education strategies that seek to increase self-efficacy certainly obtain positive and more lasting results^(9)^.

In this context, nurses play a key role in providing educational strategies aimed at promoting children’s health^(10)^, and it is essential to consider self-efficacy when developing actions with educational technologies. Thus, among the most widely used health technologies are educational videos, which help nurses and other healthcare professionals provide guidance. This technology can be used as a complementary instrument to facilitate the teaching-learning process, allowing a moment of knowledge through interaction, participation and exchange of experiences among those involved^(11)^.

Thus, the development and use of educational videos in child health provides advantages, such as the possibility of pausing, rewinding certain moments of the video and sending the link so that they can watch it again at another time. Furthermore, it is important that the scenes are close to the target audience’s reality and specificities in order to achieve the intended objectives^(12)^.

## OBJECTIVES

To develop and assess an educational video to promote self-efficacy among caregivers of NBs.

## METHODS

### Ethical aspects

The study was approved by the *Universidade da Integração Internacional da Lusofonia Afro-Brasileira* (UNILAB) Research Ethics Committee. All research participants signed the Informed Consent Form (ICF).

### Study design, period and place

This is a video development study, developed in five stages: 1) content delimitation (narrative review and situational diagnosis); 2) script content construction and validity (experts); 3) video development; 4) produced video validity (experts); and 5) apparent validity (target audience)^(13,14)^. It should be noted that the video construction followed the pre-production (synopsis, argument, script, storyboard), production and post-production phases^(15)^. The study followed the Standards for Quality Improvement Reporting Excellence 2.0 guidelines to improve healthcare quality, safety and value as well as the methodology of publications. The process of creating and assessing the educational video took place from 2019 to 2021. The scenes were recorded in the Maternal and Child Health Laboratories and other facilities of UNILAB.

### Population or sample; inclusion or exclusion criteria

In the situational diagnosis stage to identify knowledge demands, sampling was done by convenience. The sample was selected according to the following inclusion criteria: caregivers of NBs, including pregnant women, parents, and family members, who were waiting for care at health centers in Acarape and Redenção, Ceará. Caregivers of premature NBs were excluded since this population requires a more specific approach to care.

In the stages for assessing script content and assessing the video already produced, experts in neonatal and child health, information technology, video or cinematography specialists participated. All experts met at least two established requirements for participation as experts, such as having knowledge/skills acquired through experience and/or special knowledge/skills in a certain type of study on the subject^(16)^. The sample size was defined by the formula that considers the final proportion of experts in relation to a given dichotomous variable and the maximum acceptable difference of this proportion: n = Zα^2^.P.(1-P)/d^2^. Thus, Zα refers to the confidence level adopted (95%); P is the minimum proportion of individuals who agree with the relevance of concepts/scenes to the video; and d is the difference in proportion considered acceptable^(17)^. Thus, the final calculation resulted in a sample of 22 experts.

In the stage for assessing the educational video by the target audience, non-probabilistic convenience sampling was used, respecting the recommendations of authors who advise the number of 25 to 50 subjects for technology/instrument validity^(18)^. The same inclusion and exclusion criteria were adopted as in the situational diagnosis stage. Thus, 25 caregivers participated, including pregnant women, mothers and fathers of NBs.

### Study protocol

The content used in video pre-production was based primarily on a narrative review of the literature, with a view to selecting publications from the World Health Organization, official documents from the Ministry of Health, other relevant agencies and journals related to NB care. Moreover, to select the content that would be addressed in the video, a situational diagnosis was carried out through a semi-structured interview, with a view to analyzing the target audience’s main demands and doubts. The statements were transcribed and organized by the similarities of the meanings of research subjects’ discourses, considering the context of each verbal expression.

Based on these findings, the next phases of video pre-production were carried out. The synopsis summarized the video content. The plot described how the characters would act and the scenes shown in the video. The script detailed what would be produced to guide the production team during filming. It was divided into 11 scenes, distributed across 13 pages, containing descriptions of the scenes and the characters’ lines. To facilitate video script text formatting and organization, Celtx^®^ software was used, a free tool in Brazilian Portuguese that consists of a practical way of producing professional scripts^(19)^. This version of the script then went through a content validity and technical validity process.

For validity, a search for experts was conducted on the *Lattes* Platform using convenience sampling and a reference network. An invitation letter was sent via email to 49 experts, of whom 27 responded and comprised the final sample of this stage, for content validity (n=24) and technical validity (n=3), exceeding the sample initially intended (n=22).

Data collection was performed using the Google Forms^®^ tool, via email, with the ICF, script and assessment instrument. The variables assessed by both content experts and technical experts were the idea concept, dramatic construction, rhythm, characters, dramatic potential, dialogues, visual style, target audience, relevance and final result of analysis. Concerning the video objectives and relevance, these variables were assessed only by content experts. Variables such as functionality, usability and efficiency were items assessed only by technical experts. A Likert-type validity instrument with five points was also used, ranging from “very little” to “very much”, allowing each scene to be assessed based on language clarity, practical relevance and theoretical relevance.

After experts had reviewed the first draft of the script, the necessary suggested changes were made. Subsequently, a storyboard was created to detail the scenes in the form of sequential drawings, similar to a comic book. This approach allows scenes to be previewed before they are filmed, as well as providing guidance on the time required for each scene, the cast of characters and the filming environment.

The video was produced by narrating and selecting texts, figures, photos and animations. The scenes were filmed in a primary healthcare unit setting, followed by external scenes and scenes with the child. Five actors (one man, two women and one NB) were required to play the main roles, and one of the researchers acted as a nurse. The production team consisted of four professionals specialized in video production, who had the necessary professional technical equipment, such as professional cameras, microphones, tripods, lighting and appropriate screens.

After filming was completed, the video was edited in post-production, with the insertion of graphic animation resources for texts, images, soundtrack, subtitles and final review of the material. This stage was carried out by a professional in the field over a period of two months.

With the video already produced and edited, the video itself was validated by experts. In addition to validating the script, the process of validating the already recorded video by judges is essential, as it allows us to identify whether the technology is suitable for what is proposed, ensuring that different knowledge is gathered to perfect it as a valid instrument for use^(14)^. It should be noted that the video was sent to all 24 content experts who initially assessed the script; however, only seven provided feedback at this stage of video assessment. They assessed the same aspects of the script validity instrument.

Appearance validity was also carried out with the target audience of caregivers of NBs, as this stage gives greater confidence to the educational technology constructed, allowing the assessment of its effectiveness in communication and understanding for the individuals for whom the technology is intended.

Those who agreed to participate were directed to a private area, where the research objectives were explained and the two-way consent form was applied. The educational video was then played individually, and at the end, an instrument was applied to assess the video understanding. For assessment, a constructed and validated instrument^(20)^ was used that covers the following domains: understanding; attractiveness; self-efficacy; cultural acceptability; and educational material persuasiveness. In addition, a checklist was used to assess the scenes’ clarity, relevance, and degree of relevance in the educational video, which also contained a space for suggestions^(21)^.

### Analysis of results, and statistics

The data relating to situational diagnosis (initial stage), obtained through a semi-structured form, were analyzed descriptively through thematic content analysis, supporting the definition of categories for the script.

To validate the script and, subsequently, the educational video produced, the collected data were analyzed using the Statistical Package for the Social Sciences version 22.0. Item adequacy analysis was carried out using the Content Validity Index (CVI), considering a value equal to or greater than 0.80 as desirable^(22)^.

## RESULTS

In the initial stage of narrative review, the content analyzed was organized into thematic categories containing the main care that should be provided to NBs: sleep; hygiene (diaper changing, umbilical cord stump, clothing hygiene and bathing products); breastfeeding; colic care; immunizations. The situational diagnosis with the target audience confirmed these topics and added aspects such as oral hygiene, evidencing convergence between sources.

The alignment between the literature and the audience’s demands was made through thematic comparison, based on topics listed in the narrative review and the content of participants’ speeches, highlighting the recurring subjects in both stages. These topics supported the argument, the synopsis elaboration and the script structuring, which were anchored in everyday situations and transformed into short dramas, in which the characters face common challenges in caring for the baby, allowing the audience to identify with the content presented.

The video takes place in a nursing office at the primary healthcare unit, where a postpartum woman (Laura) arrives for a consultation with the unit’s nurse (Dr. Sara), a woman who has recently given birth (Laura) with her NB (Pedro) and Laura’s mother (Dona Maria), with several questions about the care that should be provided. Laura is nervous, as she finds herself confused by the instructions she has received from neighbors and family members. Throughout the video, the nurse provides instructions and demonstrates how a NB should be cared for.

The first version of the script was validated in its content by 27 experts, divided into two categories composed of child healthcare professionals (15 teachers and nine healthcare professionals) and three technical professionals. Teaching experts had between three and 14 years of experience with child health, with an average of 7.8 years. Regarding care content experts, professional experience ranged from three to 22 years, with an average of 9.4 years. As for the three technical experts, two were female, with an average of two to eight years of experience in developing educational videos.

It is worth noting that the categories assessed by experts, such as idea clarity, content relevance, coherence with objectives, dramatic potential, usability and suitability for the target audience, were defined with the aim of verifying whether the video script favors learning, promoting confidence in caring for NBs. Moreover, aspects of the narrative structure, such as dialogues, character identification and rhythm, were also considered relevant to encourage engagement and facilitate content assimilation.

Table 1 shows script content assessment by health experts (teachers and healthcare professionals) and the corresponding CVI by category.

**Table 1:** Distribution of agreement and Content Validity Index of health experts (teachers and healthcare professionals) regarding the educational video script, Redenção, Ceará, Brazil, 2021 (N=24)

Variables analyzed	n	%	I-CVI category
**Idea concept**			**0.92**
Relevant and current thematic content	24	100	
Content consistent with the video’s objective of promoting self-efficacy for newborn care	22	91.6	
The video’s objective is consistent with the reality of nursing practice	23	95.8	
The context in which the video takes place is evident from the very beginning	23	95.8	
The premises set out are correct	20	83.3	
The information is understandable	21	87.5	
The information is sufficient	16	66.6	
It meets the objectives of institutions that work with newborn care	22	91.6	
It is suitable for use by healthcare professionals	23	95.6	
The content addresses behaviors	24	100	
It proposes behavior change to viewers	22	91.6	
It proposes caregivers to feel more confident/safe in caring for newborns	24	100	
It is believed that it can improve knowledge about how to care for newborns	24	100	
**Objectives**			**0.93**
The objective is clear	24	100	
The script’s objective is consistent with the objective proposed in the research of increasing self-efficacy in newborn care	23	95.8	
The objective proposed by the script is feasible	24	100	
The number of scenes is sufficient to achieve the objective	23	95.8	
The duration is adequate to achieve the video’s objective	20	83.3	
**Dramatic construction**			**0.93**
The starting point of the script has an impact	19	79.2	
As the script develops, interest grows	24	100	
The scenes do not reflect stereotypes	16	66.6	
The video motivates/encourages the mother and caregivers to learn	24	100	
**Rhythm**			**0.91**
Each scene motivates the next	24	100	
The rhythm is tiring	20	83.3	
**Characters**			**0.88**
There is empathy with characters	23	95.8	
The presentation of characters and situations is sufficient	21	87.5	
The characters resemble mothers and caregivers in reality	22	91.6	
**Dramatic potential**			**0.83**
There is excitement	17	70.8	
There are surprises	9	37.5	
**Dialogues (dramatic time)**			**0.83**
The dialogues are natural	21	87.5	
They offer vocabulary appropriate to the video’s target audience	19	79.2	
The vocabulary uses common words	16	66.6	
The active voice style is used			
There is a conclusion	23	95.8	
If so, the conclusion met the proposed objectives	23	95.8	
**Visual style (aesthetics)**			**0.90**
The scenes reflect important aspects of newborn care	23	95.8	
**Target audience**			**0.93**
The content of interest (self-efficacy for newborn care) is directly related to the target audience (newborn caregivers)	23	95.8	
The target audience identifies with the problem presented	23	95.8	
The language is compatible with the target audience’s level of knowledge	16	66.6	
The lettering is appropriate to the target audience’s reading level	22	91.6	
**Production estimate**			**0.93**
**S-CVI/Ave**			**0.90**

*I-CVI - Item-level Content Validity Index; S-CVI/AVE - Scale-level Content Validity Index/Average.*

In addition to the above, health experts (teachers and caregivers) assessed whether the sources of self-efficacy were included in the educational video scenes, and were unanimous in considering that the script: highlights personal experiences (n=24); uses vicarious experiences, i.e., third parties as models to be followed (n=24); uses verbal persuasion (n=24); and seeks to improve psychological and emotional states, alleviating tension or anxiety in caregivers (n=24).

Thus, all category variables validated through the CVI were above 0.80 and the total CVI was 0.90, meeting the requirements for the material to be considered valid. It should be noted that experts’ suggestions that were in agreement with the literature were accepted.

Script assessment by technical communication experts and the corresponding CVI by category can be verified using Table 2.

**Table 2:** Distribution of agreement and Content Validity Index of technical experts regarding the educational video script, Redenção, Ceará, Brazil, 2021 (N=3)

Variables analyzed	n	%	I-CVI category
**Idea concept**			**0.80**
Content appropriate to the video’s objective of promoting self-efficacy for newborn care	3	100	
The idea helps learning	3	100	
The idea is accessible	3	100	
The script is useful	3	100	
The script is attractive	2	66.7	
**Dramatic construction**			**0.80**
The starting point of the script has an impact	2	66.7	
As the script develops, interest grows	2	66.7	
Sufficient number of scenes	2	66.7	
Sufficient running time	1	33.3	
Script presentation is pleasant	3	100	
**Rhythm**			**0.73**
There is a growing attention, with a dramatic upward curve	2	66.7	
The rhythm is tiring	2	66.7	
There is dynamism in the environments	2	66.7	
The ways in which the scenes are presented are appropriate	3	100	
**Characters**			**0.87**
The characters’ profile is original	3	100	
The characters’ values are consistent	3	100	
**Dramatic potential**			**0.87**
An expectation is developed	2	66.7	
**Dialogues (dramatic time)**			**0.80**
In dialogue, each intervention motivates another	3	100	
There is an acceleration of the action until the culmination of the story’s climax	2	66.7	
**Visual style (aesthetics)**			**0.80**
There are many repetitions of scenery/environment	1	33.3	
The images are adequate	2	66.7	
The overall structure is creative	2	66.7	
**Referring audience**			**0.80**
The content of interest (self-efficacy for newborn care) is directly related to the target audience (newborn caregivers)	3	100	
**Production estimate**			**0.87**
**S-CVI/Ave**			**0.81**

*I-CVI - Item-level Content Validity Index; S-CVI/AVE - Scale-level Content Validity Index/Average.*

Thus, all category variables validated through CVI had values above 0.80, with the exception of rhythm (I-CVI=0.73). Furthermore, the total CVI was 0.81, meeting the requirements for the material to be considered valid.

Among experts’ recommendations, the inclusion of information related to the content stood out, such as “the hand supporting the breast should be in a “C” shape, not in a scissor shape or on top, as this will make it difficult for the milk to come out” and “include the information that, after bathing, the NB should be dried, to avoid heat loss”. Other suggestions were to increase the father’s participation in the plot, include more scenes in different environments, reduce the total video time to make it more objective and dynamic, and reinforce dialogues that would convey greater confidence to caregivers of NBs. Experts’ suggestions were accepted. Thus, some scenes were excluded, and some lines were reduced so that the video’s total estimated time through the script changed from 35 to 24 minutes, a version that was used to prepare the storyboard to guide actors and video recording.

In addition to the above, technical experts assessed three specific aspects of their area: functionality; usability; and efficiency. Concerning functionality, two judged that the video script aims to increase caregivers’ confidence in caring for NBs, and three judged that the script is capable of generating positive results. Regarding usability, three agreed that it is easy to learn the concepts used and their applications, and that the video script allows mothers to have control of the activities presented in it, being easy to apply; two judged that the video script provides help in a clear and complete manner; and only one considered that the video script provides help without being tiring. As for efficiency, three experts considered that the number and characterization of characters meet the proposed objective, and that the discourse among characters is used efficiently and understandably to users. Two assessed that the number of scenes is consistent with the proposed time for the video, while only one considered the proposed time to be adequate for the user to learn the content and feel more confident in caring for NBs.

After video production and post-production, it was subjected to new validity by health experts (Table 3).

**Table 3:** Distribution of content experts’ educational video assessment and validity, Redenção, Ceará, Brazil, 2021 (N=7)

Variables	Excellent		Very good		Good		I-CVI
	n	%	n	%	n	%	
Idea	6	85.7	1	14.3	-	-	1.0
Objective	7	100	-	-	-	-	1.0
Dramatic construction	4	57.1	3	42.9	-	-	1.0
Rhythm	4	57.1	3	42.9	-	-	1.0
Characters	5	71.4	2	28.6	-	-	1.0
Dramatic potential	3	42.8	4	57.2	-	-	1.0
Dialogues	5	71.4	2	28.6	-	-	1.0
Visual style	6	85.7	1	14.3	-	-	1.0
Video appropriateness to the target audience	6	85.7	1	14.3	-	-	1.0
Relevance	6	85.7	1	14.3	-	-	1.0
**S-CVI/Ave**							**1.0**

*I-CVI - Item-level Content Validity Index; S-CVI/AVE - Scale-level Content Validity Index/Average.*

The validity by content experts regarding the recorded and edited educational video gave a I-CVI of 1.0 in all categories assessed and, consequently, an overall CVI of 1.0.

This same version of the video was then presented to the target audience. Twenty-five people participated in this phase of the study: pregnant women (N=23), mothers (N=1) and fathers (N=1), who were waiting for care at two primary healthcare units in Redenção and Acarape (Ceará). Table 4 shows the results of this video validity.

**Table 4:** Distribution of agreement regarding the apparent validity domains among the target audience, Redenção, Ceará, Brazil, 2021 (N=25)

Domains	n	%
**Understanding**		
I can provide information about newborn sleep from the video	23	92.0
I can mention the care that should be taken for newborn hygiene (bathing, changing diapers, cleaning the umbilical cord stump)	23	92.0
**Attractiveness**		
I wanted to watch the video until the end	22	88.0
**Cultural acceptance**		
I think something in this video bothers me	3	12.0
**Self-efficacy**		
I believe I can follow what the video teaches	23	92.0
The video motivated me to “think or act” about my health and that of my newborn	25	100
The video made me feel more confident about caring for my newborn	25	100
**Persuasion**		
I intend to follow the information in the video to care for your newborn	23	92.0
If I had to inform someone else about how to care for a newborn, I would inform them as shown in the video	24	96.0

The target audience unanimously considered the video relevant and clear. Based on the degree of relevance of each scene, the overall CVI obtained was 0.96, meaning an excellent level of agreement among participants.

## DISCUSSION

In this study, the educational video aimed to offer information on the main care provided to NBs, aiming to maximize the self-efficacy of caregivers so that they could carry out care successfully.

Self-efficacy is a construct used in numerous educational technologies and is relevant for promoting the population’s health. A randomized clinical trial found that mothers, after receiving an educational intervention based on the theory of breastfeeding self-efficacy, demonstrated a notable increase in confidence compared to the control group^(23)^.

All health experts agreed that the video adequately addressed the four sources of self-efficacy: personal experiences; vicarious experiences; verbal persuasion; and psychological and affective states. These sources are important for the expectation of self-efficacy, since they contribute to people achieving this goal^(24)^. Thus, the greater the self-efficacy, the better the personal performance for actions.

Educational video consists of a promising technology to combine both the target audience’s knowledge and self-efficacy, collaborating in the adoption of new practices as care-educational technology, promoting autonomy for subjects and culminating in NB health promotion^(25,26)^.

It is clear that educational technologies such as video are increasingly being used as an alternative for health education, as evidenced in a study on home care for premature NBs, having been a tool used in a complementary way during the guidance provided in the hospital discharge process and in home visits, presenting good acceptability by the target audience^(27)^.

The video “*Cuidando do seu bebê*” featured mother, grandmother and father as the main characters to demonstrate the importance of including the entire family in the process of caring for NBs as well as providing support to this postpartum woman. Thus, the father’s participation is sometimes considered a supporting role in the care process, which can be justified by the historical framework of caregiving being almost always a female role. However, in view of social advances, paternal support cannot be disregarded, and the father figure must be present in the strategies, since he is also responsible for the child^(28)^.

Furthermore, since the initial conception of the educational video script in this study, it was decided to carry out a situational diagnosis with the target audience. It is important to note that knowing the audience and their needs is essential to address the targeted content in accordance with reality, reducing the risk of the material becoming incomprehensible to the target audience^(29)^. Thus, during the educational video script construction and validity, care was taken to make it accessible and appropriate for the target audience by investigating information relevant to the subject in a simple, clear, and objective way through scientific literature.

Before the video recording began, the script was carefully prepared and validated by both child health experts and technical experts. It should be noted that educational videos are excellent strategies for disseminating information to lay people, but they require methodological rigor during their production^(30)^.

It is important to note that the validity process is essential after the preparation of educational materials, given the need for experts with experience in the subject to be able to assess the material and make suggestions for improvement, avoiding making superficial materials available to the population, and being an important step to increase patients’ acceptance and adherence to the use of educational materials^(31)^.

Content and technical experts responsible for validating the video had experience with the topic, which allowed them to properly assess content representativeness and relevance. Therefore, it is important to validate the educational video script, as it provides a keen perspective from people with experience in the topic, increasing the reach of the material produced and its effectiveness^(12)^.

Validity by health experts (teachers and assistants) regarding the educational video script was considered adequate for the teaching-learning process, with a total CVI of 0.90, a result similar to that of the construction and validity of other educational technologies related to NB care^(32,33)^.

The overall CVI of the script for technical experts achieved a score of 0.81, also meeting the criteria required for its use in the recording of the educational video. This result reflects the importance of the research carried out and situational diagnosis to ensure a valid and adequate script, with the purpose of recognizing the main needs and difficulties encountered by caregivers^(34)^.

In addition to validating the script, it is essential that it be modified and adjusted according to expert evaluators suggestions’ so that the video production meets the expectations and desired results. Thus, in the present study, the necessary adjustments were made to the script, and the video was recorded and edited, returning to content experts for validity so that the overall CVI went from 0.90 (script) to 1.00 (recorded video). The improvement process is crucial for advancing technology, as observed in a study that followed the stages of assessing and improving an educational video^(35)^.

In this sense, during the validity process, there was multidisciplinary participation of experts, allowing the video to bring together specialized knowledge for the topic addressed and its construction, as observed in other educational video validity studies^(27,35)^. Thus, after experts’ judgment, it was possible to identify the need to exclude scenes and speeches in order to reduce the time and ensure that the population remained attentive during the instructions. It is important to highlight that the duration and dynamics of scenes influence the interest of those who watch educational videos^(36)^.

Building and validating an educational technology, bringing it closer to the target audience from its planning, development and assessment, increases the chances of it being truly understandable and attractive to the target audience. Thus, as the overall CVI obtained in the assessment of these technologies was 0.96, it was found to be acceptable by the participating population.

Aligning the video content with scientific evidence-based recommendations and the population’s needs makes the video developed in this study an important teaching tool for training nursing teams and families and promoting NB health and well-being^(35)^.

Regarding the self-efficacy domain, all participants reported that they felt more confident in caring for their NB after watching the video. Therefore, it is important that technologies such as the one in the study consider caregivers’ self-efficacy, as they allow them to increase their confidence and greater autonomy when carrying out daily care^(37,38)^.

### Study limitations

The study had as a limitation the fact that the apparent validity was only with the target audience of only one region of Brazil, which may not be the reality obtained in other regions.

### Contributions to nursing

This educational technology is expected to be used and disseminated as a complementary tool to guidelines on home care for NBs. The educational video effectively conveys essential information in an accessible manner, thereby strengthening self-efficacy regarding infant care. Additionally, its implementation contributes to the promotion of health education carried out by nurses and other healthcare professionals and empowers the target audience when using technology. It offers a resource that expands the scope of guidelines, favors learning, and promotes autonomy for continuity of care for NBs.

## CONCLUSIONS

The development of the video “*Cuidando do seu bebê*” obtained positive results in the validity processes with experts and the target audience, giving greater prior credibility to the material that is intended to be used as a health promotion and education tool with the population. Thus, according to the validity carried out by experts and the target population, the material presents evidence of validity for use by nurses.

It is also recommended that studies be developed to assess the efficiency of this material in increasing self-efficacy in caring for NBs by caregivers.

## Data Availability

The research data are available in a repository: https://doi.org/10.48331/scielodata.FCIHVP.
