# Supplementary material for: Development and assessment of an educational video to promote self-efficacy in newborn care
Source: Rev Bras Enferm. 2025 Dec 12;78(Suppl 4):e20250101. doi: 10.1590/0034-7167-2025-0101 (PMC12707762; doi:10.1590/0034-7167-2025-0101)
Supplement: Translation

# Translation: Desenvolvimento e avaliação de vídeo educativo para promoção da autoeficácia nos cuidados com o recém-nascido

**DOI:** 10.1590/0034-7167-2025-0101pt

**Authors:** Rhaiany Kelly Lopes de Oliveira, Maria Jocelane Nascimento da Silva, Hévila Ferreira Gomes Medeiros Braga, Emília Soares Chaves Rouberte, Flávia Paula Magalhães Monteiro, Antônio Marcos de Souza Soares, Benedita Shirley Carlos Rosa, Maria Vera Lúcia Moreira Leitão Cardoso, Emanuella Silva Joventino Melo

**Affiliations:** I Universidade Federal do Ceará. Fortaleza, Ceará, Brasil; II Universidade da Integração Internacional da Lusofonia Afro-Brasileira. Redenção, Ceará, Brasil

**Keywords:** Recém-Nascido, Autoeficácia, Estudo de Validação, Recursos Audiovisuais, Enfermagem.

## Abstract

**Objetivos:**

desenvolver e avaliar vídeo educativo para a promoção da autoeficácia de cuidadores de recém-nascidos.

**Métodos:**

estudo de desenvolvimento de vídeo realizado em cinco etapas: delimitação do conteúdo (revisão narrativa e diagnóstico situacional); construção e validação do conteúdo do roteiro (*experts*); desenvolvimento do vídeo; validação do vídeo produzido (*experts*); e validação aparente (público-alvo). Para a análise dos dados, utilizou-se o Índice de Validade de Conteúdo.

**Resultados:**

o vídeo “Cuidando do seu bebê” tem duração de 24 minutos e contempla os principais cuidados domiciliares ao recém-nascido. A avaliação foi feita sob o ponto de vista de aparência e conteúdo, apresentando Índice de Validade de Conteúdo superior a 0,80 em todas as etapas de validação. O público-alvo considerou por unanimidade o vídeo relevante e claro.

**Conclusões:**

o vídeo é válido para utilização pelos profissionais de saúde como ferramenta para promover a autoeficácia de cuidadores de recém-nascidos.

## INTRODUÇÃO

O nascimento de um filho faz com que todo o núcleo familiar passe por transformações, voltando a atenção completa à criança^(1)^. Esse evento produz nos familiares um misto de emoções e dificuldades com a adaptação para sua nova rotina, exigindo o desenvolvimento de habilidades e segurança nos cuidados com o recém-nascido (RN)^(2,3)^.

Sabe-se ainda que o componente neonatal prevalece com a maior parcela de óbitos no que diz respeito à mortalidade infantil geral. De acordo com os dados divulgados pelo Fundo das Nações Unidas, em 2022, cerca de 2,3 milhões dos óbitos infantis ocorreram no primeiro mês de vida^(4)^. Segundo o Ministério da Saúde, em 2019, o Brasil registrou uma taxa alarmante de 67,9 óbitos infantis por 1.000 nascidos vivos, com destaque nas regiões Norte (16,6) e Nordeste (15,2)^(5)^.

Esta realidade alarmante de saúde está transformando a abordagem da prática de enfermagem, uma vez que os enfermeiros são desafiados diariamente a se concentrar na promoção da saúde e na educação das comunidades. Essa mudança vai além da abordagem tradicional centrada nos cuidados restauradores com pacientes com problemas de saúde agudos, estendendo-se para áreas como saúde infantil no âmbito da atenção primária^(6)^.

Destaca-se, portanto, a relevância de aprimorar as ações de educação em saúde, fornecendo orientações abrangentes e divulgando informações durante os encontros entre gestantes e profissionais de saúde^(7)^, bem como puérperas e familiares do RN. Nesse contexto, estratégias de educação em saúde que considerem a mãe e a família como protagonistas dos cuidados oferecidos ao RN são essenciais, especialmente no contexto domiciliar.

A mãe precisa acreditar em sua capacidade de cuidar da criança para que de fato ela possa executar tais cuidados de maneira efetiva. Essa crença trata-se da autoeficácia, caracterizada como a confiança na habilidade pessoal de executar com êxito comportamentos ligados à parentalidade, desempenhando um papel central nesse cenário desafiador^(8)^. Para tanto, deve-se considerar que as estratégias de educação em saúde que buscam elevar a autoeficácia, certamente, obtêm resultados positivos e mais duradouros^(9)^.

Nesse contexto, o enfermeiro tem papel de destaque para oportunizar estratégias educacionais voltadas para a promoção da saúde da criança^(10)^, sendo essencial considerar a autoeficácia durante o desenvolvimento de ações com tecnologias educacionais. Dessa forma, entre as tecnologias mais utilizadas no contexto da saúde, tem-se os vídeos educativos, os quais auxiliam os enfermeiros e demais profissionais de saúde durante as orientações. Essa tecnologia pode ser utilizada como instrumento complementar para facilitar o processo de ensino-aprendizagem, permitindo um momento de conhecimento mediante a interação, participação e troca de experiências entre os envolvidos^(11)^.

Assim, o desenvolvimento e uso de vídeos educativos no contexto da saúde da criança proporciona vantagens, tais como a possibilidade de pausar, retroceder em alguns momentos do vídeo e envio do *link* para que possam assistir novamente em outro momento. Ademais, é importante que as cenas se aproximem da realidade e especificidades do público-alvo para assim alcançar os objetivos pretendidos^(12)^.

## OBJETIVOS

Desenvolver e avaliar um vídeo educativo para a promoção da autoeficácia de cuidadores de RNs.

## MÉTODOS

### Aspectos éticos

O estudo foi aprovado pelo Comitê de Ética em Pesquisa da Universidade da Integração Internacional da Lusofonia Afro-Brasileira (UNILAB). Todos os participantes da pesquisa assinaram o Termo de Consentimento Livre e Esclarecido (TCLE).

### Desenho, período e local de estudo

Trata-se de estudo de desenvolvimento de vídeo, desenvolvido em cinco etapas: 1) delimitação do conteúdo (revisão narrativa e diagnóstico situacional); 2) construção e validação do conteúdo do roteiro (*experts*); 3) desenvolvimento do vídeo; 4) validação do vídeo produzido (*experts*); e 5) validação aparente (público-alvo)^(13,14)^. Salienta-se que a construção do vídeo seguiu as fases de pré-produção (sinopse, argumento, roteiro, *storyboard*), produção e pós-produção^(15)^. O estudo seguiu as diretrizes da *Standards for QUality Improvement Reporting Excellence 2.0* para aprimorar a qualidade, segurança e valor dos cuidados em saúde, bem como a metodologia das publicações. O processo de construção do vídeo educativo e avaliação ocorreu de 2019 a 2021. As cenas foram gravadas nos Laboratórios de Saúde Materno Infantil e demais dependências da UNILAB.

### População ou amostra; critérios de inclusão ou exclusão

Na etapa de diagnóstico situacional para identificação de demandas de conhecimentos, a amostragem se deu por conveniência. A amostra foi selecionada conforme os seguintes critérios de inclusão: cuidadores de RNs, sejam eles gestantes, pais e familiares, que estavam em espera de atendimento nos centros de saúde dos municípios de Acarape e Redenção, Ceará. O critério de exclusão considerou cuidadores de RNs prematuros, uma vez que, para este público, os cuidados exigem um olhar mais específico.

Nas etapas de avaliação do conteúdo do roteiro e avaliação do vídeo já produzido, contou-se com a participação de *experts* em saúde neonatal e infantil, e tecnologia da informação, especialistas em vídeos ou cinematografia. Todos os *experts* atingiram no mínimo dois requisitos estabelecidos previstos para a participação como especialistas, como possuir conhecimento/habilidade adquirido pela experiência e/ou conhecimento/habilidade especial em determinado tipo de estudo na temática^(16)^. O tamanho amostral foi definido pela fórmula que considera a proporção final de *experts* em relação a uma determinada variável dicotômica e à diferença máxima aceitável desta proporção: n = Zα^2^.P.(1-P)/d^2^. Assim, Zα refere-se ao nível de confiança adotado (95%); P é a proporção mínima de indivíduos que concordam com a pertinência dos conceitos/cenas ao vídeo; e d é a diferença de proporção considerada aceitável^(17)^. Assim, o cálculo final resultou em uma amostra de 22 *experts*.

Na etapa de avaliação do vídeo educativo pelo público-alvo, utilizou-se a amostragem não probabilística por conveniência, respeitando as recomendações de autores que aconselham o número de 25 a 50 sujeitos para validação de tecnologia/instrumentos^(18)^. Adotaram-se os mesmos critérios de inclusão e exclusão da etapa de diagnóstico situacional. Assim, houve a participação de 25 cuidadores, como gestantes, mães e pais de RNs.

### Protocolo do estudo

O conteúdo utilizado na pré-produção do vídeo foi fundamentado primeiramente em revisão narrativa da literatura, com vistas a selecionar publicações da Organização Mundial da Saúde, documentos oficiais do Ministério da Saúde, outros órgãos de interesse e em periódicos relacionados à temática de cuidados com os RNs. Além disso, para seleção do conteúdo que seria abordado no vídeo, realizou-se diagnóstico situacional por meio de entrevista semiestruturada, com vistas a analisar as principais demandas e dúvidas do público-alvo. As falas foram transcritas, organizadas pelas similitudes dos significados dos discursos dos sujeitos da pesquisa, considerando o contexto de cada expressão verbal.

Diante dos achados, seguiu-se para as próximas fases da pré-produção do vídeo. Na sinopse, realizou-se o resumo do conteúdo do vídeo. No argumento, descreveu-se como seria a ação dos personagens e das cenas evidenciadas no vídeo. O roteiro foi o detalhamento do que seria produzido para guiar a equipe de produção das filmagens. Este foi dividido em 11 cenas, distribuídas em 13 páginas, contendo descrição das cenas e falas dos personagens. Para facilitar a formatação e organização do texto do roteiro do vídeo, foi utilizado o *software* Celtx^®^, uma ferramenta em português e gratuita, consistindo em uma forma prática de produção de roteiros profissionais^(19)^. Em seguida, esta versão do roteiro passou por um processo de validação de conteúdo e validação técnica.

Para validação, foi feita uma busca pelos *experts* na Plataforma Lattes com amostragem por conveniência e por rede de referência. Foi enviada uma carta convite via e-mail para 49 *experts*, dos quais 27 deram retorno e compuseram a amostra final desta etapa, sendo para validação de conteúdo (n=24) e validação técnica (n=3), superando-se a amostra inicialmente pretendida (n=22).

A coleta de dados foi realizada por meio da ferramenta do *Google Forms* ^®^, via e-mail, com o TCLE, o roteiro e o instrumento de avaliação. As variáveis avaliadas tanto pelos *experts* no conteúdo quanto pelos *experts* técnicos foram conceito da ideia, construção dramática, ritmo, personagens, potencial dramático, diálogos, estilo visual, público-alvo, relevância e resultado final da análise. Quanto aos objetivos e à relevância do vídeo, estas variáveis foram avaliadas apenas pelos *experts* no conteúdo. Já as variáveis funcionalidade, usabilidade e eficiência foram itens avaliados apenas pelos *experts* técnicos. Utilizou-se ainda um instrumento de validação do tipo Likert com cinco pontos, variando de “pouquíssimo” a “muitíssimo”, permitindo avaliar cada cena a partir da clareza da linguagem, pertinência prática e relevância teórica.

Após a apreciação da primeira versão do roteiro pelos *experts*, foram realizadas as devidas modificações sugeridas. Em seguida, construiu-se o *storyboard* para detalhamento das cenas em forma de desenhos sequenciais, semelhantes a uma história em quadrinhos. Essa abordagem permite pré-visualizar as cenas antes de serem gravadas, além de orientar sobre o tempo necessário para cada uma, o elenco de personagens e o ambiente das filmagens.

Para a produção do vídeo, seguiu-se com a narração, seleção de textos, figuras, fotos e animações. As cenas foram realizadas para um cenário da Unidade de Atenção Primária à Saúde, seguidas de cenas externas e cenas com a criança. Foi necessária a participação de cinco atores (um homem, duas mulheres e um RN) nos papéis principais, e uma das pesquisadoras atuou como enfermeira. A equipe de produção foi composta por quatro profissionais especialistas em produção de vídeos, os quais dispunham do equipamento técnico profissional necessário, tais como câmeras profissionais, microfones, tripés, iluminação e anteparos apropriados.

Após a conclusão das filmagens, seguiu-se para a pós-produção para a edição do vídeo, com a inserção dos recursos de animação gráfica de textos, imagens, trilha sonora, legendas e revisão final do material. Esta etapa foi realizada por um profissional da área no período de dois meses.

Com o vídeo já produzido e editado, procedeu-se à validação do vídeo propriamente dito junto aos *experts*. Além de validar o roteiro, o processo de validação do vídeo já gravado junto a juízes é fundamental, visto que permite identificar se a tecnologia está adequada ao que propõem, garantindo reunir diferentes saberes para aperfeiçoá-lo como instrumento válido para uso^(14)^. Salienta-se que o vídeo foi enviado para todos os 24 *experts* de conteúdo que avaliaram o roteiro inicialmente; no entanto, apenas sete deram retorno nesta fase de avaliação do vídeo. Estes avaliaram os mesmos aspectos do instrumento de validação do roteiro.

Procedeu-se ainda à validação de aparência junto ao público-alvo de cuidadores de RN, pois esta etapa confere maior confiança à tecnologia educativa construída, permitindo avaliar a eficácia na comunicação e compreensão para os indivíduos aos quais a tecnologia se destina.

Aqueles que aceitaram participar foram direcionados para um local reservado, onde foram explicados os objetivos da pesquisa e aplicado o TCLE em duas vias. Em seguida, procedeu-se à reprodução do vídeo educativo de forma individual, e ao término, foi aplicado um instrumento para avaliar a compreensão sobre o vídeo. Para a avaliação, utilizou-se um instrumento construído e validado^(20)^ que contempla os seguintes domínios: compreensão; atratividade; autoeficácia; aceitabilidade cultural; e persuasão do material educativo. Além disso, utilizou-se *checklist* para avaliação quanto à clareza, à relevância e ao grau de relevância das cenas do vídeo educativo, contendo também um espaço destinado para sugestões^(21)^.

### Análise dos resultados e estatística

Os dados referentes ao diagnóstico situacional (etapa inicial), obtidos por meio de formulário semiestruturado, foram analisados de forma descritiva por meio da análise de conteúdo temática, subsidiando a definição das categorias para o roteiro.

Para a validação do roteiro e, posteriormente, do vídeo educativo produzido, os dados coletados foram analisados no programa estatístico *Statistical Package for the Social Sciences*, versão 22.0. A análise da adequação dos itens se deu por meio do Índice de Validade de Conteúdo (IVC), considerando-se valor igual ou superior a 0,80 como desejável^(22)^.

## RESULTADOS

Na etapa inicial de revisão narrativa, o conteúdo analisado foi organizado em categorias temáticas contendo os principais cuidados que devem ser prestados aos RNs: sono; higiene (troca de fraldas, coto umbilical, produtos para higiene das roupas e banho); aleitamento materno; cuidados com cólicas; imunizações. O diagnóstico situacional junto ao público-alvo confirmou essas temáticas e acrescentou aspectos como higiene oral, evidenciando convergência entre as fontes.

O alinhamento entre a literatura e as demandas do público foi feito por meio da comparação temática, a partir dos tópicos elencados na revisão narrativa e do conteúdo das falas dos participantes, destacando os assuntos recorrentes nas duas etapas. Esses temas subsidiaram o argumento, a elaboração da sinopse e a estruturação do roteiro, os quais foram ancorados em situações cotidianas e transformados em pequenos dramas, nos quais os personagens enfrentam desafios comuns no cuidado com o bebê, possibilitando identificação do público com os conteúdos apresentados.

O cenário do vídeo acontece em um consultório de enfermagem da Unidade de Atenção Primária à Saúde, onde chega para consulta com a enfermeira da unidade (Dr^a^. Sara), uma puérpera (Laura) com seu RN (Pedro) e a mãe de Laura (Dona Maria), com várias dúvidas sobre os cuidados que devem ser prestados. Laura está nervosa, pois vê-se confusa nas orientações que recebeu de vizinhos e familiares. Ao longo do vídeo, a enfermeira realiza as orientações e demonstrações de como os cuidados devem ser realizados com o RN.

A primeira versão do roteiro foi validada em seu conteúdo por 27 *experts*, divididos em duas categorias compostas por profissionais de saúde da criança (sendo 15 docentes e nove profissionais assistenciais) e três profissionais técnicos. Os *experts* docentes possuíam variação de três a 14 anos de experiência com a temática saúde da criança, com média de 7,8 anos. Em relação aos *experts* de conteúdo assistenciais, a experiência profissional variou de três a 22 anos, com média de 9,4 anos. Quanto aos três *experts* técnicos, dois eram do sexo feminino, com média de dois a oito anos de experiência em desenvolvimento de vídeo educativo.

Destaca-se que as categorias avaliadas pelos *experts*, como clareza da ideia, relevância do conteúdo, coerência com os objetivos, potencial dramático, usabilidade e adequação ao público-alvo, foram definidas com o objetivo de verificar se o roteiro do vídeo favorece a aprendizagem, promovendo a confiança nos cuidados com os RNs. Além disso, aspectos da estrutura narrativa, como os diálogos, a identificação dos personagens e o ritmo, também foram considerados relevantes para estimular o engajamento e facilitar a assimilação do conteúdo.

A avaliação do conteúdo do roteiro pelos *experts* de saúde (docentes e assistenciais) e o IVC correspondente por categoria podem ser verificados por meio da Tabela 1.

**Tabela 1:** Distribuição da concordância e do Índice de Validade de Conteúdo dos *experts* de saúde (docentes e assistenciais) quanto ao roteiro vídeo educativo, Redenção, Ceará, Brasil, 2021 (N=24)

Variáveis analisadas	n	%	I-CVI da categoria
**Conceito da ideia**			**0,92**
Conteúdo temático relevante e atual	24	100	
Conteúdo coerente com objetivo do vídeo de promover a autoeficácia para cuidados com o recém-nascido	22	91,6	
Objetivo do vídeo é coerente com a realidade da prática de enfermagem	23	95,8	
O contexto em que o vídeo se passa é evidente desde o primeiro momento	23	95,8	
As premissas expostas estão corretas	20	83,3	
As informações são compreensíveis	21	87,5	
As informações são suficientes	16	66,6	
Atende aos objetivos de instituições que trabalham com cuidados com o recém-nascido	22	91,6	
É adequado para ser usado por profissionais de saúde	23	95,6	
O conteúdo aborda comportamentos	24	100	
Propõe ao telespectador mudança de comportamento	22	91,6	
Propõe aos cuidadores sentirem-se mais confiantes/seguros nos cuidados com o recém-nascido	24	100	
Acredita que poderá melhorar o conhecimento sobre como cuidar do recém-nascido	24	100	
**Objetivos**			**0,93**
O objetivo é evidente	24	100	
O objetivo do roteiro é coerente com o objetivo proposto na pesquisa de elevar a autoeficácia nos cuidados com o recém-nascido	23	95,8	
O objetivo proposto pelo roteiro é factível	24	100	
O número de cenas é suficiente para o alcance do objetivo	23	95,8	
O tempo de duração é adequado para atingir o objetivo do vídeo	20	83,3	
**Construção dramática**			**0,93**
O ponto de partida do roteiro tem impacto	19	79,2	
Com o desenvolvimento do roteiro o interesse cresce	24	100	
As cenas não refletem estereótipos	16	66,6	
O vídeo motiva/estimula a mãe e cuidadores a aprenderem	24	100	
**Ritmo**			**0,91**
Cada cena motiva a próxima	24	100	
O ritmo é cansativo	20	83,3	
**Personagens**			**0,88**
Existe empatia com as personagens	23	95,8	
A apresentação das personagens e situações são suficientes	21	87,5	
As personagens lembram mães e cuidadores da realidade	22	91,6	
**Potencial dramático**			**0,83**
Existe emoção	17	70,8	
Existem surpresas	9	37,5	
**Diálogos (tempo dramático)**			**0,83**
Os diálogos têm naturalidade	21	87,5	
Oferecem vocabulário adequado à população para a qual o vídeo se destina	19	79,2	
O vocabulário utiliza palavras comuns	16	66,6	
O estilo de voz ativa é utilizado			
Há conclusão	23	95,8	
Se sim, a conclusão atendeu aos objetivos propostos	23	95,8	
**Estilo visual (estética)**			**0,90**
As cenas refletem aspectos importantes dos cuidados com o recém-nascido	23	95,8	
**Público-alvo**			**0,93**
O conteúdo de interesse (autoeficácia para os cuidados com o recém-nascido) tem relação direta com o público-alvo (cuidadores de recém-nascido)	23	95,8	
Existe identificação do público-alvo com a problemática exposta	23	95,8	
A linguagem está compatível com o nível de conhecimento do público-alvo	16	66,6	
O conteúdo a ser lido (*lettering*) é adequado ao nível de leitura do público-alvo	22	91,6	
**Estimativa de produção**			**0,93**
**S-CVI/Ave**			**0,90**

*I-CVI - Item-level Content Validity Index; S-CVI/AVE - Scale-level Content Validity Index/Average.*

Além do exposto, os *experts* de saúde (docentes e assistenciais) avaliaram se as fontes de autoeficácia estavam contempladas nas cenas do vídeo educativo, tendo sido unânime ao considerarem que o roteiro: ressalta experiências pessoais (n=24); utiliza experiências vicárias, ou seja, terceiros como modelos a serem seguidos (n=24); utiliza a persuasão verbal (n=24); e busca melhorar estados psicológicos e afetivos, aliviando tensões ou a ansiedade do cuidador (n=24).

Dessa forma, todas as variáveis de categorias validadas por meio do IVC deram acima de 0,80 e o IVC total deu 0,90, satisfazendo ao exigido para que o material seja considerado válido. Salienta-se que as sugestões dos *experts* que estavam de acordo com a literatura foram acatadas.

A avaliação do roteiro pelos *experts* técnicos de comunicação e o IVC correspondente por categoria podem ser verificados por meio da Tabela 2.

**Tabela 2:** Distribuição da concordância e do Índice de Validade de Conteúdo dos *experts* técnicos quanto ao roteiro vídeo educativo, Redenção, Ceará, Brasil, 2021 (N=3)

Variáveis analisadas	n	%	I-CVI da categoria
**Conceito da ideia**			**0,80**
Conteúdo adequado ao objetivo do vídeo de promover autoeficácia para os cuidados com o recém-nascido	3	100	
A ideia auxilia a aprendizagem	3	100	
A ideia é acessível	3	100	
O roteiro é útil	3	100	
O roteiro é atrativo	2	66,7	
**Construção dramática**			**0,80**
O ponto de partida do roteiro tem impacto	2	66,7	
Com o desenvolvimento do roteiro o interesse cresce	2	66,7	
Número de cenas suficiente	2	66,7	
Tempo de duração suficiente	1	33,3	
Apresentação do roteiro é agradável	3	100	
**Ritmo**			**0,73**
Existe uma atenção crescente, com curva dramática ascendente	2	66,7	
O ritmo é cansativo	2	66,7	
Há dinamismo dos ambientes	2	66,7	
As formas de apresentação das cenas são adequadas	3	100	
**Personagens**			**0,87**
O perfil das personagens é original	3	100	
Os valores das personagens têm consistência	3	100	
**Potencial dramático**			**0,87**
É desenvolvida uma expectativa	2	66,7	
**Diálogos (tempo dramático)**			**0,80**
No diálogo cada intervenção motiva outra	3	100	
Há aceleração da ação até o ponto culminante do clímax da história	2	66,7	
**Estilo visual (estética)**			**0,80**
Existem muitas repetições de cenário/ambiente	1	33,3	
As imagens são adequadas	2	66,7	
A estrutura geral é criativa	2	66,7	
**Público-alvo**			**0,80**
O conteúdo de interesse (autoeficácia para os cuidados com o recém-nascido) tem relação direta com o público-alvo (cuidadores de recém-nascido)	3	100	
**Estimativa de produção**			**0,87**
**S-CVI/Ave**			**0,81**

*I-CVI - Item-level Content Validity Index; S-CVI/AVE - Scale-level Content Validity Index/Average.*

Dessa forma, todas as variáveis de categorias validadas por meio do IVC tiveram valores acima de 0,80, com exceção do ritmo (I-CVI=0,73). Além disso, o IVC total deu 0,81, satisfazendo ao exigido para que o material seja considerado válido.

Entre as recomendações dos *experts,* destacou-se a inclusão de informações relacionadas ao conteúdo, como “a mão de apoio do seio deve ser em formato de “C”, não em formato de tesoura ou por cima, pois isso vai dificultar a saída do leite” e “inserir a informação de que, após o banho, o RN deve ser enxugado, para evitar perda de calor”. Foram também sugeridos a ampliação da participação do pai no enredo, a inclusão de mais cenas em ambientes variados, a redução do tempo total do vídeo para torná-lo mais objetivo e dinâmico, e o reforço de diálogos que transmitisse maior confiança aos cuidadores do RN. As sugestões dos *experts* foram acatadas. Dessa forma, algumas cenas foram excluídas, e algumas falas, reduzidas, de forma que o tempo total estimado do vídeo por meio do roteiro mudou de 35 para 24 minutos, versão essa que foi utilizada para a elaboração do *storyboard* para orientar os atores e a gravação do vídeo.

Além do citado, os *experts* técnicos avaliaram três quesitos específicos de sua área: funcionalidade; usabilidade; e eficiência. Quanto à funcionalidade, dois julgaram que o roteiro do vídeo se propõe a elevar a confiança de cuidadores para cuidar do RN, e três julgaram que o roteiro é capaz de gerar resultados positivos. Em relação à usabilidade, três concordaram que é fácil de aprender os conceitos utilizados e suas aplicações, e que o roteiro do vídeo permite que a mãe tenha controle das atividades nele apresentadas, sendo fácil de aplicar; dois julgaram que o roteiro do vídeo fornece ajuda de forma clara e completa; e apenas um considerou que o roteiro do vídeo fornece ajuda sem ser cansativo. Acerca da eficiência, três *experts* consideraram que o número e a caracterização dos personagens atendem ao objetivo proposto, e que o discurso entre os personagens é usado de forma eficiente e compreensível à clientela. Dois avaliaram que o número de cenas está coerente com o tempo proposto para o vídeo, enquanto que apenas um considerou o tempo proposto adequado para que o usuário aprenda o conteúdo e se sinta mais confiante para cuidar do RN.

Após a produção e pós-produção do vídeo, este foi submetido a uma nova validação por parte dos *experts* de saúde (Tabela 3).

**Tabela 3:** Distribuição da avaliação e validação dos *experts* de conteúdo acerca do vídeo educativo, Redenção, Ceará, Brasil, 2021 (N=7)

Variáveis	Excelente		Muito bom		Bom		I-CVI
	n	%	n	%	n	%	
Ideia	6	85,7	1	14,3	-	-	1,0
Objetivo	7	100	-	-	-	-	1,0
Construção dramática	4	57,1	3	42,9	-	-	1,0
Ritmo	4	57,1	3	42,9	-	-	1,0
Personagens	5	71,4	2	28,6	-	-	1,0
Potencial dramático	3	42,8	4	57,2	-	-	1,0
Diálogos	5	71,4	2	28,6	-	-	1,0
Estilo visual	6	85,7	1	14,3	-	-	1,0
Adequação do vídeo ao público-alvo	6	85,7	1	14,3	-	-	1,0
Relevância	6	85,7	1	14,3	-	-	1,0
**S-CVI/Ave**							**1,0**

*I-CVI - Item-level Content Validity Index; S-CVI/AVE - Scale-level Content Validity Index/Average.*

A validação dos *experts* de conteúdo acerca do vídeo educativo gravado e editado conferiu I-CVI de 1,0 em todas as categorias avaliadas e, consequentemente, IVC global de 1,0.

Com isso, essa mesma versão do vídeo foi apresentada ao público-alvo. Participaram dessa fase do estudo 25 pessoas, gestantes (N=23), mães (N=1) e pai (N=1), que estavam esperando atendimento em duas Unidades de Atenção Primária à Saúde de Redenção e Acarape (Ceará). Os resultados desta validação do vídeo constam na Tabela 4.

**Tabela 4:** Distribuição da concordância quanto aos domínios de validação aparente junto ao público-alvo, Redenção, Ceará, Brasil, 2021 (N=25)

Domínios	n	%
**Compreensão**		
Consigo informar sobre o sono do recém-nascido a partir do vídeo	23	92,0
Posso citar os cuidados que devem ser realizados para higiene (banho, troca de fraldas, limpeza do coto umbilical) do recém-nascido	23	92,0
**Atratividade**		
Tive vontade de assistir ao vídeo até o fim	22	88,0
**Aceitação cultural**		
Considero que algo nesse vídeo me incomoda	3	12,0
**Autoeficácia**		
Acredito que posso seguir o que o vídeo ensina	23	92,0
O vídeo me motivou a “pensar ou agir” sobre a minha saúde e a do recém-nascido	25	100
O vídeo fez com que eu me sentisse mais confiante para cuidar do recém-nascido	25	100
**Persuasão**		
Eu pretendo seguir as informações do vídeo para cuidar do seu recém-nascido	23	92,0
Caso eu tenha que informar alguma outra pessoa a como prestar cuidados a um recém-nascido, informaria conforme mostra o vídeo	24	96,0

O público-alvo considerou por unanimidade o vídeo relevante e claro. A partir do grau de relevância de cada cena, o IVC global obtido foi 0,96, significando excelente nível de concordância entre os participantes.

## DISCUSSÃO

Neste estudo, o vídeo educativo teve como proposta oferecer informações sobre os principais cuidados prestados ao RN, visando maximizar a autoeficácia de cuidadores para que pudessem realizar os cuidados com êxito.

A autoeficácia trata-se de construto utilizado em inúmeras tecnologias educativas, sendo relevante para a promoção da saúde da população. Ensaio clínico randomizado constatou que mães, após receberem intervenção educativa fundamentada na teoria da autoeficácia sobre amamentação, demonstraram um aumento notável de confiança em comparação ao grupo controle^(23)^.

Todos os *experts* de saúde concordaram que o vídeo abordou de maneira adequada as quatro fontes de autoeficácia: experiências pessoais; experiências vicárias; persuasão verbal; e estados psicológicos e afetivos. Tais fontes são importantes para a expectativa de autoeficácia, uma vez que contribuem para as pessoas alcançarem este objetivo^(24)^. Assim, quanto maior a autoeficácia, melhor será o desempenho pessoal para as ações.

O vídeo educativo consiste em uma tecnologia promissora para aliar tanto conhecimentos quanto autoeficácia do público-alvo, colaborando para a adoção de novas práticas enquanto tecnologia cuidativo-educacional, promovendo a autonomia dos sujeitos e culminando com a promoção da saúde do RN^(25,26)^.

É notório que as tecnologias educacionais como o vídeo se tornam cada vez mais empregadas como uma alternativa para a educação em saúde, conforme foi evidenciado em estudo sobre cuidados domiciliares com RN prematuros, tendo sido uma ferramenta utilizada de forma complementar durante as orientações fornecidas no processo de alta hospitalar e em visitas domiciliares, apresentando uma boa aceitabilidade por parte do público-alvo^(27)^.

O vídeo “Cuidando do seu bebê” trouxe entre as personagens mãe, avó e pai como protagonistas para demonstrar a importância de incluir toda a família no processo de cuidado com o RN, bem como para dar apoio a essa puérpera. Assim, a participação do pai é, por vezes, considerada coadjuvante no processo do cuidado, o que pode se justificar por todo o arcabouço histórico do cuidar ser quase sempre um papel feminino. No entanto, diante dos avanços sociais, o suporte paterno não pode ser desconsiderado, e nas estratégias, precisa estar presente a figura do pai, uma vez que ele também é responsável pela criança^(28)^.

Ademais, desde a concepção inicial do roteiro do vídeo educativo neste estudo, optou-se pela realização de um diagnóstico situacional junto ao público-alvo. Ressalta-se que conhecer o público e suas necessidades é fundamental para abordar os conteúdos voltados, conforme a realidade, diminuindo o risco de o material se tornar incompreensível para o público-alvo^(29)^. Assim, no decorrer da construção e validação do roteiro do vídeo educativo, teve-se o cuidado de torná-lo acessível e adequado ao público-alvo, investigando, mediante a literatura científica, informações pertinentes ao assunto de forma simples, clara e objetiva.

Antes mesmo de se iniciar a gravação do vídeo, elaborou-se cuidadosamente o roteiro do mesmo, o qual passou pelo processo de validação tanto com *experts* de saúde da criança quanto com *experts* técnicos. Reforça-se que vídeos educativos são excelentes estratégias para a divulgação de informações para pessoas leigas, mas precisam de um rigor metodológico durante a sua produção^(30)^.

Ressalta-se que o processo de validação é essencial após a elaboração de materiais educativos, visto a necessidade de que *experts* com experiência no assunto possam avaliar o material e fazer sugestões para o seu aperfeiçoamento, evitando disponibilizar para a população materiais superficiais, sendo uma etapa relevante para elevar a aceitação e adesão dos pacientes na utilização de materiais educativos^(31)^.

Os *experts* de conteúdo e técnicos responsáveis pela validação do vídeo possuíam experiências com a temática, o que lhes permitia avaliar adequadamente a representatividade e relevância de conteúdo. Assim, é importante a validação do roteiro do vídeo educativo, pois oportuniza um olhar aguçado de pessoas com experiência no tema, aumentando o alcance do material produzido e sua eficácia^(12)^.

A validação pelos *experts* de saúde (docentes e assistenciais) quanto ao roteiro do vídeo educativo foi considerada adequada ao processo de ensino-aprendizagem, com o IVC total de 0,90, resultado similar ao de construção e validação de outras tecnologias educacionais relacionadas ao cuidado com o RN^(32,33)^.

O IVC total do roteiro para os *experts* técnicos alcançou uma pontuação de 0,81, atendendo também aos critérios exigidos para sua utilização na gravação do vídeo educativo. Este resultado reflete a importância da pesquisa realizada e do diagnóstico situacional para a garantia de um roteiro válido e adequado, com o propósito de reconhecer as principais necessidades e dificuldades encontradas pelos cuidadores^(34)^.

Além de validar o roteiro, é essencial que este seja modificado e ajustado conforme as sugestões dos *experts* avaliadores, para que a produção do vídeo alcance as expectativas e resultados desejados. Assim, no presente estudo, procederam-se aos ajustes necessários no roteiro, sendo o vídeo gravado e editado, retornando à validação dos *experts* de conteúdo, de forma que o IVC global passou de 0,90 (roteiro) para 1,00 (vídeo gravado). A realização do processo de melhoria é crucial para o aprimoramento da tecnologia, sendo observado no estudo, o qual seguiu etapas semelhantes de avaliação e aperfeiçoamento de um vídeo educativo^(35)^.

Nesse sentido, durante o processo de validação, houve a participação multiprofissional de *experts*, permitindo que o vídeo reunisse saberes especializados para o tema abordado e sua construção, sendo observado em outros estudos de validação de vídeo educativo^(27,35)^. Assim, após julgamento dos *experts,* foi possível identificar a necessidade de excluir cenas e falas, a fim de reduzir o tempo e garantir que a população permanecesse atenta durante as orientações. É importante destacar que a duração e a dinâmica das cenas influenciam o interesse de quem assiste vídeos educativos^(36)^.

Construir e validar uma tecnologia educacional, aproximando-a do público-alvo desde o seu planejamento, passando pelo desenvolvimento e avaliação, aumenta as chances de ser realmente compreensível e atraente ao público ao qual se destina. Assim, como o IVC global obtido na avaliação desses obteve valor de 0,96, verificou-se uma aceitabilidade por parte da população participante.

Ao alinhar o conteúdo do vídeo com as recomendações baseadas em evidências científicas, sugeridas pelos *experts*, e as necessidades da população, o vídeo desenvolvido neste estudo se torna uma importante ferramenta de ensino para capacitar a equipe de enfermagem e as famílias, promovendo a saúde e o bem-estar dos RNs^(35)^.

Com relação ao domínio de autoeficácia, todos os participantes referiram que se sentiram mais confiantes em cuidar do seu RN após assistir ao vídeo. Logo, é importante que tecnologias como a do estudo considerem a autoeficácia dos cuidadores, pois permitem aumentar sua confiança e uma maior autonomia durante a realização dos cuidados do cotidiano^(37,38)^.

### Limitações do estudo

O estudo possuiu como limitação o fato de a validação aparente ter sido apenas com o público-alvo de somente uma região do Brasil, o que pode não ser a realidade obtida em outras regiões.

### Contribuições para a área da enfermagem

Espera-se que esta tecnologia educacional seja utilizada e divulgada como uma ferramenta complementar às orientações sobre os cuidados domiciliares ao RN. O vídeo educativo facilita a transmissão de informações essenciais de forma acessível e didática, fortalecendo a autoeficácia quanto aos cuidados com o bebê. Além disso, sua implementação contribui tanto para a valorização da educação em saúde, realizada por enfermeiros e demais profissionais de saúde, quanto para o empoderamento do público-alvo ao utilizar a tecnologia, oferecendo um recurso que amplia o alcance das orientações e favorece a aprendizagem e autonomia para a continuidade do cuidado com o RN.

## CONCLUSÕES

O desenvolvimento do vídeo “Cuidando do seu bebê” obteve resultados positivos nos processos de validação com *experts* e com o público-alvo, conferindo maior credibilidade prévia ao material que se deseja utilizar como ferramenta de promoção e educação em saúde com a população. Dessa forma, de acordo com a validação realizada pelos *experts* e população-alvo, o material apresenta evidências de validade para utilização pelos enfermeiros.

Recomenda-se ainda o desenvolvimento de estudos para avaliar a eficiência deste material em elevar a autoeficácia nos cuidados com os RNs pelos cuidadores.

## Data Availability

Os dados de pesquisa estão disponíveis em repositório: https://doi.org/10.48331/scielodata.FCIHVP.
